# Trachoma and Ocular Chlamydial Infection in the Era of Genomics

**DOI:** 10.1155/2015/791847

**Published:** 2015-09-03

**Authors:** Tamsyn Derrick, Chrissy h. Roberts, Anna R. Last, Sarah E. Burr, Martin J. Holland

**Affiliations:** Department of Clinical Research, Faculty of Infectious Tropical Diseases, London School of Hygiene and Tropical Medicine, London WC1E 7HT, UK

## Abstract

Trachoma is a blinding disease usually caused by infection with *Chlamydia trachomatis (Ct)* serovars A, B, and C in the upper tarsal conjunctiva. Individuals in endemic regions are repeatedly infected with *Ct* throughout childhood. A proportion of individuals experience prolonged or severe inflammatory episodes that are known to be significant risk factors for ocular scarring in later life. Continued scarring often leads to trichiasis and in-turning of the eyelashes, which causes pain and can eventually cause blindness. The mechanisms driving the chronic immunopathology in the conjunctiva, which largely progresses in the absence of detectable *Ct* infection in adults, are likely to be multifactorial. Socioeconomic status, education, and behavior have been identified as contributing to the risk of scarring and inflammation. We focus on the contribution of host and pathogen genetic variation, bacterial ecology of the conjunctiva, and host epigenetic imprinting including small RNA regulation by both host and pathogen in the development of ocular pathology. Each of these factors or processes contributes to pathogenic outcomes in other inflammatory diseases and we outline their potential role in trachoma.

## 1. Introduction

Sightsavers International estimates that every 15 minutes a person loses sight as a result of trachoma [1]. As such, trachoma remains the world's leading infectious cause of blindness despite significant efforts to control and eliminate the disease [2]. Trachoma is currently considered endemic in 51 countries worldwide and only seven formerly endemic countries have reached target elimination thresholds [2]. The Alliance for the Global Elimination of Blinding Trachoma has set the goal of 2020 for the elimination of trachoma. The aim is to control trachoma through the implementation of surgery for trichiasis, antibiotics to treat infection, facial cleanliness, and environmental improvements to reduce transmission (SAFE). Currently 31 trachoma endemic countries implement SAFE, which is effective in controlling trachoma if well conducted. Azithromycin is the antibiotic of choice used in mass drug administration (MDA) programmes for trachoma control. There are additional beneficial effects of azithromycin MDA, including reduced all-cause mortality [3] and potential to reduce clinical disease through its anti-inflammatory properties [4]. There remains a need to pursue vaccine development as there are circumstances when SAFE is poorly effective and there is uncertainty about its universal application. The lack of randomized controlled trials examining the effectiveness of the F and E components for the interruption of transmission, alongside the historical lack of molecular laboratory tools able to identify transmission events, raises questions on the basic understanding of their effectiveness. Additional concerns with the A component include the long-term use of antibiotics in populations where MDA has failed to control disease [5], introduction of resistance in other bacterial species [6], and the continued progression of scarring and trichiasis in populations where MDA has been implemented [7]. It is also not currently understood whether effective mass treatment leads to arrested immunity and it is unclear what impact the elimination of ocular chlamydial exposure in childhood might exert later in adolescent and adult urogenital disease. Chlamydiae can reside in the gastrointestinal tract in the absence of clinical disease and this has led to the suggestion that azithromycin treatment failures (at least in urogenital disease) may be because gastrointestinal Chlamydiae are refractory to azithromycin treatment and can act as a source for autoinoculation [8, 9]. A vaccine offering effective long-term protection against disease in both ocular and urogenital chlamydial disease therefore remains desirable.

**Figure 1:**
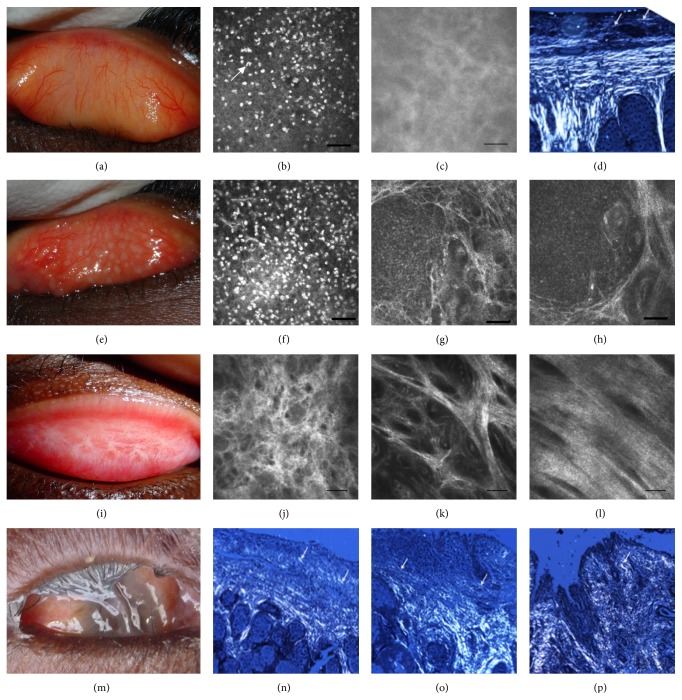
Images from a normal healthy eye (a–d) and from individuals with follicular trachoma (e–h), trachomatous scarring (i–l), and trichiasis and progressive scarring (m–p). (a), (e), (i), and (m) are photographs of the tarsal conjunctiva showing normal appearance (a), papillary inflammation and follicles (e), bands of trachomatous scarring (i), and extreme trichiasis and corneal opacity (m). (b), (c), (f), (g), (h), (j), (k), and (l) are* in vivo* confocal microscopy images of the tarsal conjunctiva at various depths (the bar represents 50 *μ*m). A moderate number of inflammatory nuclei are present in the subepithelium of a healthy eye (b), whereas a higher number are present in trachomatous inflammation (f). Follicles can be seen in (g) and (h). The connective tissue of the healthy conjunctiva is amorphous (c), whereas successive grades of trachomatous scarring are seen as a heterogeneous clumpy appearance (j), defined tissue bands that make up <50% of the scan area (k), and defined bands that make up >50% of the scan area (l). (d) and (n–p) are histological images of tissue scarring using polarized light (original magnification ×100). In the healthy conjunctiva, collagen fibers are parallel (arrows) with the surface epithelium (d), whereas progressive disorganization of this appearance is observed in scarring (n–p). Images are kindly provided with permission from Matthew Burton and Victor Hu and are adapted from Hu et al. 2011 [11] and Hu et al. 2013 [12].

Trachoma is initiated by infection of the tarsal conjunctiva with the intracellular bacteria* Chlamydia trachomatis* (*Ct*). There are a number of classification systems for the clinical signs of trachoma. Under the WHO simplified grading system, the presence of five or more follicles on the conjunctival surface is classified as trachomatous inflammation follicular (TF).* Ct* infection is independently associated with TF (OR * *= * *11.2 (95% CI 6.9–18.1) [10]), although this value varies between populations depending on disease sign prevalence and becomes disassociated from TF once prevalence is low. Repeated* Ct* infection in endemic communities can trigger chronic conjunctival inflammation (trachomatous inflammation intense, TI) in some individuals, causing conjunctival fibrosis (trachomatous scarring, TS). Progressive fibrosis may lead to entropion, inward turning, or misdirected lashes (trachomatous trichiasis, TT), all of which abrade the corneal surface. This abrasive damage may lead to corneal opacity (CO) and blindness.  Figure 1 shows reflective* in vivo* confocal microscopy scans, histology sections, and photographs of the tarsal conjunctiva that illustrate the changes in tissue architecture that occur in the different stages of trachomatous disease.

The human trachoma vaccine trials that took place in the 1960s concluded that some short-term strain-specific protection from infection was induced, amidst concerns that pathology was exacerbated in some cases, supported by data from monkey models [13]. The data from these large placebo-controlled trials has been recently reinterpreted in the context of current grading systems and our current knowledge of disease pathogenesis. Only trial III in The Gambia recorded evidence of conjunctival scarring. Two doses of prophylactic vaccination made from two live strains in mineral oil were given three weeks apart to children aged 0–4 years. There was no protection from active trachoma; however the vaccinated group had a reduced prevalence of scarring disease two years later [14]. When all three Gambian trials are reviewed in the context of what is now known about disease pathogenesis, vaccine-induced exacerbation of disease may not be a significant concern, raising hopes that current vaccine formulations may be successful [15, 16].

## 2. Immunopathology of Trachoma

Despite extensive research, the mechanistic link between chronic inflammation and progressive scarring remains elusive [7]. The scarring observed in both trachoma and urogenital disease is thought to be a result of the host response to infection rather than a direct effect of the bacteria. Defining the features of protective versus pathogenic immunological responses in chlamydial disease (reviewed in [17]) is crucial to the understanding of disease and for efficacious vaccine design.

Currently, two models have been put forward to explain the immunopathogenesis of chlamydial disease: the immunological and cellular paradigms. The traditional immunological paradigm implicates the adaptive immune system. Initial studies on chlamydial infection found that repeated infection exacerbated inflammation and that inflammatory pathology continued in the absence of* Ct*. At first, a delayed type hypersensitivity (DTH) reaction triggered by a chlamydial antigen was hypothesized. The chlamydial heat shock protein 60 (HSP60) was considered a candidate antigen to trigger DTH but was not found to exacerbate pathology when tested in a guinea-pig model [18]. Antibodies to HSP60 predicted a 2-3-fold higher risk of* Ct* pelvic inflammatory disease (PID) in humans and higher levels of antibodies specific to HSP60 were found in women with the most severe forms of urogenital disease [19]. Likewise antibodies to HSP60 were associated with tubal factor infertility (TFI) and inflammatory trachoma [20, 21]; however it remains unclear whether these antibodies cause pathology or are a result of a greater number or more severe episodes of infection. More recent screening of sera from trichiasis patients and controls detected differential patterns of antibody recognition for a number of* Ct* antigens; however HSP60 responses were not significantly different [22].

Previous research demonstrated that individuals with scarring trachoma had Th2 patterns of cytokine expression [23], whereas those with strong Th1 responses efficiently cleared infection [24]. This opposed the DTH theory and suggested that a Th2 response either was ineffectual at clearing infection or led to enhanced pathology and that CD4+ Th1 IFN*γ* responses were a key element of protective immunity in trachoma. Both CD8+ and CD4+ T cell infiltrates are associated with the conjunctival follicles that are characteristic of TF; however CD4+ T cells are thought to outnumber CD8+ [25]. NK cells have more recently been identified as a major early source of IFN*γ* in response to* Ct* [26]. Despite the primary function of CD8+ T cells in defense against intracellular pathogens, data from murine models previously found a limited role for CD8+ T cells in anti-*Ct* immunity [27]. Recent evidence in the macaque model of trachoma challenges this theory and suggests that depletion of CD8+ T cells can abrogate protective immunity [28]. Characterization of the roles of each of these cell types in chronic scarring trachoma has proved difficult to address, however, largely due to the natural history of scarring disease.

Stephens suggested an alternative model of disease [29] in which epithelial cells at the infection site play a central role as mediators of the innate immune response. Epithelial cells infected with* Ct* secrete a number of proinflammatory cytokines and growth factors, such as IL-8, GRO*α*, IL-1*α*, IL-6, and granulocyte-macrophage colony-stimulating factor (GM-CSF). The secretion of these cytokines is delayed compared to the kinetics of secretion upon infection with other invasive bacteria and persists throughout the 2–4-day developmental cycle of* Ct*. The secretion of IL-1*α* by infected cells was shown to upregulate proinflammatory cytokine production by neighboring uninfected cells, promoting a strong inflammatory response [30]. Lymphocytes of the adaptive immune system are attracted to the infection site by the chemotactic gradient and subsequent infections include memory populations that amplify the response.* Ct* infected cells also secreted the profibrotic cytokine IL-11, a member of the IL-6 family [31, 32]. Recent evidence from a primary-like polarized epithelial cell model showed that IL-11 was secreted preferentially from the basolateral membrane and IL1ra from the apical membrane in* Ct* infected cells [33]. The T cell chemokines RANTES and IP10 were also downregulated in productively* Ct* infected cells, suggesting that* Ct* attempts to avoid stimulation of the immune response in order to maintain its intracellular niche, potentially leading to longer infections and chronic pathology [33]. Recent genome-wide scale array studies from clinical samples support Stephens's hypothesis. Expression profiling of children in The Gambia with active trachoma versus healthy controls revealed a strong upregulation of the innate immune response [34]. Along with the expected infiltration of neutrophils, lymphocytes, and the inflammatory cytokine pathways consistent with histopathology [35], the natural killer (NK) cell response was also enhanced. A transcriptome study conducted in Ethiopian adults with either trachomatous trichiasis (TT) or TT with inflammation (TTI) versus healthy controls found that despite less than 1%* Ct* infection prevalence, markers of ongoing subclinical inflammation and tissue remodeling were evident in the TT group and were more pronounced in the TTI group [36]. These markers were consistent with an activated and proinflammatory epithelium. There was a lack of evidence for a profibrotic Th2 response and a very limited Th1 response. Subsequent targeted quantitative-PCR studies have reemphasized the role of the innate immune response in trachoma in both children and adults [37, 38]. These studies found antimicrobial peptides psoriasin (*S100A7*),* DEFb4A*, and* SAA1*, inflammatory cytokines* IL17*,* IL1β*, and* TNF*, inflammatory chemokines (*CCL18* and* CXCL5*),* MMPs*, and* CTGF* were upregulated in trachomatous disease. Increased* IL17* expression was highlighted as characteristic of active trachoma and is thought to coordinate the proinflammatory response [37].

Overall these studies suggest that clearance of* Ct* infection requires an appropriate acute Th1 response with IFN*γ* and CD8+ T cells. They also support the case for the central role of epithelial cells in ongoing trachomatous inflammation and pathogenesis that leads to TT. This would imply that neither the cellular nor the immunological paradigm can fully explain disease but that active cross talk between cellular (innate epithelial cell responses) and immunological (adaptive immunity) systems is required.

## 3. Host Genetic Association Studies and Trachoma

The assumed link between immune driven inflammation and conjunctival scarring and its blinding sequelae led to the first genetic association studies which focused on immune response genes. Immune response genes located within the Major Histocompatibility Complex (MHC) drive the adaptive cellular response and initial studies focused on the association of trachomatous scarring disease with alleles in HLA class I and class II loci. The intracellular nature of chlamydial infection suggested that class I restricted CD8+ T cell responses would be important in controlling infection and that these might be related to later scarring disease. MHC class II loci would be expected to contribute by defining the profile of anti-*Ct* antibody responses and cytokines via CD4+ class II restricted T cells.

The first reported genetic association study in trachoma in Gambians [39] found evidence that a specific HLA class I serological determinant of the HLA-A2 supertype (HLA-A28) was associated with scarring trachoma (OR = 1.88, *P* = 0.046) and observed that approximately 26% of scarring cases had HLA-A28, compared to 16% of controls. HLA-A28 is made up of at least 3 molecular subtypes (HLA-A^*∗*^68:01, HLA-A^*∗*^68:02, and HLA-A^*∗*^69:01), of which HLA-A^*∗*^68:02 was thought to be the most frequent allele in Gambians. In a subset analysis of a limited number of samples from the same study, molecular typing showed that the HLA-A^*∗*^68:02 allele was strongly associated with scarring trachoma (OR = 3.14, CI 1.32–7.44, and *P* = 0.009). No alleles of either of the MHC class II genes, HLA-DRB1 or HLA-DQB1, were found to be associated with scarring. Antibody responses to chlamydial HSP60 were positively correlated with the allele HLA-DRB1^*∗*^07:01 (OR = 2.6, *P* = 0.02) and negatively correlated with both HLA-DQB1^*∗*^03:01 (OR = 0.42, *P* < 0.001) and HLA-DQB1^*∗*^05:01 (OR = 0.55, *P* = 0.046) [40]. Later studies also linked HLA class II alleles to anti-*Ct* antibody prevalence and quantity in the clinical context of sexually transmitted* Ct* infections [41, 42].

It had previously been shown that synthetic peptides based on chlamydial sequences (MOMP and HSP60) could elicit* HLA-B8* and* HLA-B35* restricted CD8+ CTL responses in peripheral blood of individuals from trachoma endemic regions [43]. In an attempt to link the observed* HLA-A* ^*∗*^68:02 association to an effector mechanism, a follow-up study [44] investigated whether* HLA-A* ^*∗*^68:02 restricted CD8+ CTLs or IFN*γ* producing cells were associated with* Ct* specific immune responses in a small number of scarring cases and disease-free controls. Peptides from 3 chlamydial antigens that were predicted to bind HLA-A^*∗*^68:02 were used to stimulate cells but no chlamydia-peptide specific responses were detected. This led the authors to suggest either that the peptides that were chosen did not represent natural epitopes or that* HLA-A* ^*∗*^68:02 restricted CD8+ T cells were not important mediators in ocular disease. An alternative experimental approach and one that did not require* in vitro* restimulation used* HLA-A2*-MOMP tetramers and found that increased frequencies of CD8+ tetramer positive cells were coincident with current ocular* Ct* infection and longer durations of infection [45]. Effector functions of these cells were not investigated but it was noted that the tetramers used to identify cells also bound to the TCR of HLA-A28 restricted T cells. This supported a model under which the HLA-A^*∗*^68:02 allele might contribute to scarring disease via prolonged or chronic ocular infections.

Outside of trachoma, antichlamydial CD8+ MHC class I restricted cells have been clearly demonstrated in murine models [46] and in humans exposed to urogenital infections [47]. Recently there has been a resurgence of interest in the importance of CD8+ MHC class I restricted T cells, following work in a murine model of urogenital disease [48] and in vaccinated monkeys [49]. Following vaccination with the plasmid-free attenuated strain of* Ct*, monkeys with a common MHC class II haplotype (M1) were protected from virulent ocular challenge. Subsequent work found that protection was mediated through CD8+ T cell recognition of soluble chlamydial antigens [28] and not through class II restricted CD4+ T cells. Most recently, an immune-proteomic screening approach in mice showed that only a small percentage (~3%) of the* Ct* proteome is processed for presentation by MHC class II molecules.* Ct* proteins that were processed were the expected immunodominant proteins such as MOMP, PmpE, PmpF, and PmpG and were presented on a wide range of I-A and I-E MHC haplotypes [50]. The immunogenicity of these proteins was also subject to both MHC class II selection and chlamydial peptide sequence diversity. Overall, this suggests that studies of sufficient size and power to account for the high degree of polymorphism and small effect size are required to further investigate HLA class II disease susceptibility. Overall, reports of associations between human MHC polymorphisms and chlamydial infection and disease have been inconsistent and have not identified a specific causal pathway. Collectively these studies do suggest an important but complex role for the MHC in chlamydial host resistance and disease.

Following the early MHC class I and class II genetic association studies in Gambians, a trachoma-HLA association study was conducted in Omanis using a serologically based typing system. This found some evidence of association between TT and the HLA class II DR16 and DR53 antigens [51]. Further studies in Tanzanians [52] found that trichiasis was less common in women carrying* HLA-DRB1* ^*∗*^11 alleles (OR = 0.48, CI 0.26–0.90, and *P* = 0.02) and more common in those carrying* HLA-B* ^*∗*^07 (OR = 3.26, CI 1.42–7.49, and *P* = 0.004) or* HLA-B* ^*∗*^08 (OR = 5.12, CI 1.74–15.05, and *P* = 0.001). More recently, a much larger study in Gambian families also highlighted the association between* HLA-B* ^*∗*^08:01 alleles and scarring [53], but analysis based on current knowledge of the HLA system would suggest that HLA-B^*∗*^08:01 could be a proxy marker for the HLA-C epitope of the Killer Cell Immunoglobulin-like Receptors (KIRs). This epitope is defined by a dichotomous amino acid polymorphism at position 80 of the HLA-C heavy chain [54]. HLA-C alleles carry Asn^80^ and are designated C1 whilst those with Lys^80^ are C2. The HLA-C KIR epitope determines which KIR can bind the ligand, with KIR2DL1 binding to HLA-C2 and both KIR2DL2 and KIR2DL3 binding to HLA-C1 [55–57]. KIR are a polymorphic family of membrane-bound immune-receptors found on natural killer (NK) cells [58–60], where they are usually considered to control the licensing [61] and responsiveness [62, 63] of the NK cytotoxic immune response. KIRs are also present on NK-T cells and T cells [64, 65], though the functional contribution of KIRs to T cell reactivity is relatively less well studied [66, 67]. The finding that HLA-C2 was a significant predictor [53] of increased risk of scarring in Gambian families (OR_C1/C2_ = 2.29, *P*_C1/C2_ = 0.0061∣OR_C2/C2_ = 3.97, *P*_C2/C2_ = 0.0004) further suggested a role for NK cells in trachomatous scarring. Epistatic effects were observed since KIR (Chromosome 19) genotype modified the risk of scarring depending on HLA-C (Chromosome 6) genotype; HLA-C2 showed additive gene dosage effects and individuals carrying both* KIR2DL2* and* KIR2DL3* had the highest relative risk (OR_C1/C2_ = 2.33, *P*_C1/C2_ = 0.1∣OR_C2/C2_ = 5.95, *P*_C2/C2_ = 0.0025). NK cells are likely to be important in the initial host response to ocular challenge, since it has been shown that they are the main cellular source of IFN*γ* in response to* in vitro* elementary body stimulation [26]. There was no evidence in the study of scarring trachoma in Gambian families that the previously identified* HLA-A* ^*∗*^68:02 allele was associated with early scarring (*P* = 0.27).

MHC class I and class II association studies were also extended into class III loci. A polymorphism in the tumour necrosis factor (*TNF*) promoter region was found to associate with scarring under an additive model (OR_A/G_ = 1.59, OR_A/A_ = 3.4, and *P* = 0.03) [68]. Whilst scarring disease was also associated with increased detection of TNF protein in tear fluid (OR_add_ = 2.5, *P* = 0.013), TNF levels in the tear fluid did not associate with the genotypes of the TNF-308 polymorphism. A later study of trichiasis patients and controls found an association between TNF-308A and disease, which was significant under a dominant, rather than additive, genetic model (OR_dom_ = 1.52, *P* = 0.016) [69]. These later data also suggested evidence for heterozygote advantage (OR_A/G_ = 1.48, *P* = 0.048∣OR_A/A_ = 1.11, *P* = 0.12) in the context of a simple genotype model; however adjustments for multiple testing were not included. Lymphocytes from TNF-308A individuals had increased TNF secretion* in vitro* in response to challenge with* Ct* elementary bodies. Curiously, the TNF-308A polymorphism has also been identified [70] as a protective factor in trichiasis (OR_A/G_ = 0.45 [0.25–0.81], *P* = 0.008∣OR_A/A_ = 0.19 [0.04–1.08], *P* = 0.062) in a study that also identified homozygosity at a lymphotoxin alpha (LTA) polymorphism (LTA252 OR_A/A_ = 0.25 [0.09–0.63], *P* = 0.004), heterozygosity at an Interleukin 9 polymorphism (IL9-T113M OR_C/T_ = 0.25 [0.1–0.64], *P* = 0.004), and heterozygosity at the vascular cell adhesion molecule 1 (VCAM1, OR = 0.47 [0.25–0.86], *P* = 0.015) as protective factors for trichiasis [70]. The same authors suggested that epistasis between the polymorphisms was potentially key to unraveling the host genetic background of trachoma. TT risk increased substantially (OR = 13.5, *P* = 0.001) when the TNFA (-308G), VDR (intron G), IL4R (50 V), and ICAM1 (56 M) polymorphisms were considered as genetic constellations under a “logic regression” model [70], but the number of samples contributing to this analysis was small (controls *n* = 232 and TT *n* = 135).

**Figure 2:**
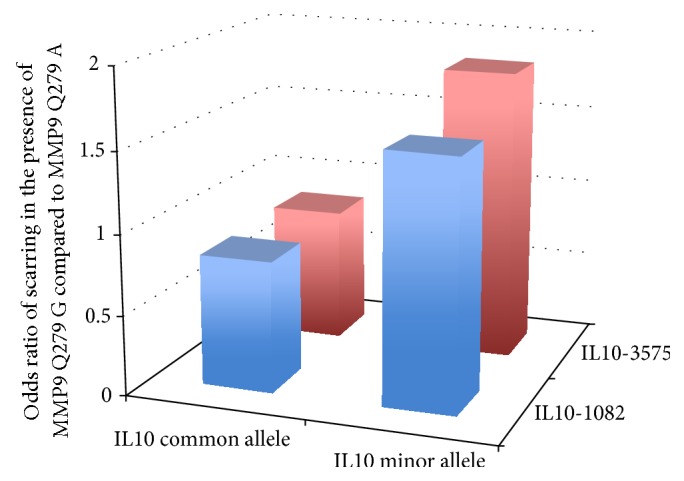
Evidence for MMP9-IL10 epistasis in Gambians with trachomatous scarring. The protective effect of* MMP9* allele (Q279R) is modulated by host genetic background at the* IL10* locus such that protective effects of the G allele are lost in the presence of either of 2 minor frequency risk alleles (*IL10*-1082C or* IL10*-3575A). The interaction between these nonallelic genes (or risk genotypes) has a dominant effect over other combinations. The interaction between risk genotypes was examined by conditional likelihood ratio tests (LRT) (main effects) log p/1 − p = a + b (SNP1) + c (SNP2). Interaction terms were defined as Log p/1 − p = a + b(SNP1) + c(SNP2) + d(SNP1*∗*SNP2) and when significant identified statistical epistasis. This approach was applied to 651 Gambian case-control pairs of TS. The* MMP9* Q279R and* IL10*-3575 loci showed strong evidence for statistical interaction affecting risk of TS (LRT *χ* ^2^ = 7.23, *P* = 0.007). Carriers of the (protective)* MMP9* Q279R G allele who also had the* IL10*-3575A minor allele were at significantly increased risk of TS (OR = 1.83 (1.06–3.19)) when compared to subjects with the* IL10*-3575 T allele (common allele) (OR = 0.84 (0.69–1.02)). The* IL10*-1082 C minor allele in combination with the* MMP9* Q279R G allele had an increased risk of TS (OR = 1.51 (1.02–2.24)) relative to IL10-1082 C in the presence of the* MMP9* Q279R A allele (OR = 0.82 (0.67–1.01)) (LRT *χ* ^2^ = 7.53, *P* = 0.006 for the interaction between the* IL10*-1082 and* MMP9* risk alleles). Interaction between* IL10*-1082 and* MMP9* Q279R affects risk despite the null single SNP main effect for* IL10*-1082 [71]. The individually protective* MMP9* Q279R G allele was therefore associated with an increased risk of scarring in the presence of* IL10* risk alleles (*IL10*-1082C or* IL10*-3575A minor alleles) and a decreased risk in the presence of common* IL10* (protective) alleles. Similar modelling at other loci previously investigated in this cohort (IFN*γ*-1616, +3234; LTA -252, +77; IkBL -63; IL-8 -251; GM-CSF2 27348, 27438) showed significant or marginally significant evidence for two-way interactions, at the genotype or allelic level, with the MMP9 Q279R SNP. Some of these SNPs are in high LD and therefore not all the hypotheses tested are independent. The existence both of LD between loci and of potential biological interdependence between loci raises methodological difficulties in correction for multiple testing. We did not attempt any correction for multiple testing: and therefore a contribution of chance to these results is difficult to exclude as we point out in the main text. Comparing main and additional two-way epistatic effects in the final model suggested that the inclusion of interaction terms improved the fit of the model to the data, so that the final best model included both main and epistatic effects. For TS this model suggested that two-way interactions of* MMP9*-Q279R with* IFNγ*-1616,* IFNγ*+3234,* IL10*-1082,* IL8*-251,* LTA*+77,* LTA*-252, and* IkBL*-63 improved the fit of the model (data courtesy of Natividad, Mabey, Holland, and Bailey).

A series of studies used SNP genotypes and linkage disequilibrium data to predict haplotypes and then carried out haplotype based association tests in genes including* IFNγ* [71],* IL8* [72],* IL10* [71, 73], haptoglobin (*HP*),* GM-CSF2* [72], and matrix metalloproteinase 9 (*MMP9*) [74]. The* MMP9* gene is consistently differentially expressed between cases and controls of several stages of trachomatous disease [34, 36]. A study of the* IL8/CSF2* loci [72] also noted some evidence for epistatic interactions between polymorphisms in* MMP9* and each of the* IL8* and* CSF2* genes. Other epistatic interactions were also found in the same dataset, in particular, an* MMP9-IL10* interaction (Figure 2). In the analysis of a cohort of 651 scarring case-control pairs, main effects for the* IL10-1082* allele were null; however interaction between* IL10*-1082 and* MMP9* Q279R contributed to risk. The protective effects of the* MMP9* Q279R G allele were transformed to risk in the copresence of a SNP (*IL10*-1082) tagging the trachoma risk haplotype (*IL10*-3575A~*IL10*-1082C~*IL10*-592G~*IL10*+5009G). This allele or haplotype is associated with higher levels of* IL10* transcription [73]. This may suggest that premature or excessive downregulation of MMP9 activity by IL-10 ultimately increases risk. Each of these studies suffers from limited sample size and large numbers of parallel statistical tests under several different genetic models, whilst sometimes also considering multiple phenotypes (i.e., scarring and trichiasis). Consequently the results should be interpreted in the context of potential type I and type II errors.

The candidate gene approach has helped reinforce current lines of investigation but has yet to resolve the paradigm of the cellular versus immunological hypotheses or generate new avenues of investigation into the basic biology of chlamydial ocular disease. A genome-wide association scan (GWAS) is an alternative to the candidate gene method and provides an opportunity to generate new hypotheses in an unbiased manner. GWAS requires much larger sample sizes than have been previously described in trachoma and chlamydial urogenital disease and has its own limitations, particularly when applied to samples from African populations [75]. There are also relatively few GWAS in infectious disease [76]. The West African malaria GWAS is perhaps a cautionary example [77]. The study showed that SNP markers in very close proximity to the well-characterized causal SNP (rs334) of the “sickle-cell trait” protective effect failed to reach genome-wide significance, even though the study was well powered. This was largely due to the low degree of linkage disequilibrium (LD) between SNPs in African populations. The rs334 SNP, when directly genotyped, was highly significant (*P* = 1.3 × 10^−28^), whilst the best marker in the GWAS SNPs (*P* = 3.9 × 10^−7^) did not reach genome-wide significance. The use of more densely populated GWAS SNP arrays and imputation methods can increase the likelihood of finding significant SNPs [78], but imputation is essentially dependent on LD structures and the imputation of rs334 in the malaria GWAS was far less effective (*P* = 4.5 × 10^−14^) than direct genotyping of the locus [77]. GWAS in African populations are less likely to directly identify SNPs or alleles in association with disease than equivalently sized studies in other populations. Therefore the utility of a trachoma GWAS will most likely be in prioritizing the best candidates and biologically plausible pathways. The small effects of numerous polymorphisms in functionally linked networks potentially have much greater contribution to disease when tested* en masse*. Several groups are currently pursuing genome-wide approaches to the understanding of the host genetics of chlamydial diseases, either in human clinical populations of pelvic inflammatory disease [79], trachoma [80], and advanced recombinant inbred mouse models [81] or in “cellular GWAS,” which uses* in vitro* infection of extensively genotyped host cell lines [82, 83]. The potential power of a pathway-focus in genome-wide studies is evident from the preliminary reports in trachoma [80] and a mouse model system [81], both of which emphasize the importance of G-protein coupled receptor signaling pathways in disease processes relating to chlamydial infections.

## 4. Chlamydial Genomics and Pathogenesis

Initial single gene, multilocus sequence typing, and subsequent whole genome sequencing (WGS) of chlamydial species have provided insight into the processes involved in chlamydial pathogenesis. The first fully sequenced* Chlamydia trachomatis* genome was a laboratory reference strain (D/UW-3/Cx) passaged in the laboratory for more than 30 years after its initial isolation from a cervical swab in 1965. The genome was completed by shotgun sequencing using highly purified* Ct* genomic DNA obtained by bulk culture to produce sufficient genomic DNA to generate the M13 linker-adaptor library. More than 28,000 sequencing reactions using dye-labeled primers and almost 4,700 dye-terminator reactions were required to complete the genome [84]. Ten to twelve years later, the first ocular chlamydial genomes were fully sequenced prior to the wide scale introduction of next generation sequencing (NGS) [85, 86]. Comparative genomic analysis of these and subsequent chlamydial isolates demonstrated overall similarity in gene content and order across biovars and between chlamydial species [87–90]. This high level synteny is correlated with the extent of evolutionary divergence [90]. Application of NGS technologies has rapidly advanced and expanded our understanding of chlamydial comparative and evolutionary dynamics. Recent WGS analysis demonstrated that there is extensive recombination within and between biovars [91] (also noted in other studies [92, 93]) and that there is evidence for genetic exchange and recombination within the cryptic plasmid [91]. This has challenged our knowledge of chlamydial diversity and highlighted the implications of recombination in pathogenesis. Other significant advances including genetic manipulation of Chlamydiae [94, 95], mutagenesis studies [95–98], and axenic culture [99] facilitate the study of chlamydial metabolism and physiology in the context of genetic studies. Novel presequencing enrichment techniques such as immunomagnetic separation and DNA-baiting techniques (SureSelect) have removed technical barriers to obtaining WGS data directly from clinical specimens [100, 101]. Moreover, the ability to obtain WGS data directly from clinical samples without the need for culture obviates the problem of* in vitro* propagation and subsequent loss of genomic diversity reflecting differences between* in vitro* and* in vivo* evolutionary environments, as has been demonstrated in* Ct* [102–104] and herpesvirus species (Varicella Zoster Virus and* Cytomegalovirus*) [105, 106]. This allows the study of genetic variants associated with disease, including those under positive selective pressure through interaction with the host. Direct and targeted deep resequencing has also enabled discovery of mixed infections within seed stocks of cultures and from clinical isolates [104, 107]. Putative virulence factors identified through WGS analysis and* in vitro* and animal studies include the polymorphic outer membrane protein family (Pmps) [84, 108–110], type three secretion system (T3SS) and effectors [111–113], genes involved in tryptophan [114–116] and glycogen [94, 117] biosynthesis, members of the* incA* [118, 119] and phospholipase-D [120, 121] families, genes from the heat shock protein family [122, 123], the chlamydial cytotoxin [124], and plasmid-encoded genes implicated in the regulation of chromosomal virulence factors [97, 125, 126].

Pmps appear to fulfill several biological functions, but the full extent of their role is not entirely clear. These exported bacterial proteins have been shown to function as autotransporters within the chlamydial outer membrane [127–129]. Several chlamydial Pmps have been detected on the chlamydial elementary body surface [127]. Some are strongly immunogenic and elicit a proinflammatory response [127]. They may play a role in virulence regarding modulation of inflammation and adherence to and invasion of host cells [129].

The chlamydial plasticity zone (PZ) is the site of extensive genomic variation between the chlamydial serovars [88, 90]. The PZ contains a toxin whose function is thought to involve GTPase inactivation and guanylate binding protein neutralization resulting in interferon gamma (IFN*γ*) resistance [124, 130, 131]. IFN*γ* stimulation of cells* in vitro* and* in vivo* induces transcription of genes that cooperate to eliminate intracellular bacteria, suggesting that inhibition of this mechanism may contribute to the pathogenesis of chlamydial disease [131]. The genomic organisation of the cytotoxin varies such that oculogenital strains have a single gene with a large deletion and lymphogranuloma venereum (LGV) strains lack the gene entirely, whilst the closely related* C. muridarum* (*Cm*) has three intact copies [132, 133]. This most likely reflects the IFN*γ* evasion mechanism employed by* Cm* in the mouse compared with that employed by urogenital strains in humans [133]. Since ocular strains lack a functional* trp* operon and only have a partial toxin, how they evade IFN*γ* is not fully understood. The membrane attack complex/perforin protein (CT153) and members of the phospholipase-D (PLD) family are also encoded within the PZ [90]. The PZ PLDs are present in* Ct* [120] and other Chlamydiaceae [87, 134, 135]. These proteins are known to modify lipids and membranes and may be secreted and localize to the inclusion membrane [121]. Reactivation of* Ct* from an IFN*γ*-induced persistent state is blocked by primary alcohols (potent PLD inhibitors), implying a role for PZ PLDs in this process [136].

Genomic analysis has shown that many Inc proteins are produced by each chlamydial species and several are produced early in development. Inc proteins, which are T3SS effector proteins, may be important antigens in cell-mediated immune responses following infection. IncA has been defined as an effector protein proposed to mediate inclusion fusion in* Ct* [118, 119]. IncA proteins exhibit an innate ability to form vesicles and enter the membrane of intracellular compartments [137]. A recent study of the Inc-human “interactome” using affinity-purification mass spectrometry identified that 38/58 Incs interacted with human proteins [138]. Many of the host targets were also targeted by viral proteins implying conserved mechanisms of intracellular pathogenesis. IncE bound to components of the retromer, which restricted chlamydial growth [138].

Work on the chlamydial T3SS [112] in combination with the advent of WGS demonstrated that a family of chlamydial proteins that localize to the inclusion membrane during intracellular growth were a candidate T3SS [111]. This system is important in bacterial pathogenesis and is essential for virulence of a number of bacterial species [139]. In most of these pathogens, the structural and effector genes are located on distinct pathogenicity islands [140]. In Chlamydiae, these genes are scattered across a number of operons and use a sigma factor for constitutive gene expression, suggesting that additional activators or repressors are required if components of the T3SS are developmentally regulated [113]. Given the intracellular existence of Chlamydiae, these T3 secreted products are likely to be important in pathogenesis.

The chlamydial plasmid has been shown to function as a virulence factor in animal models [49, 141]. Phenotypic differences vary between plasmid-cured and wild-type strains with respect to infectivity, glycogen accumulation, induction of inflammation, and activation of toll-like receptor pathways [117, 142]. Plasmid deletion mutagenesis studies showed that deletion of the plasmid-encoded* pgp4* gene results in an* in vitro* phenotype identical to that of a plasmid-free strain [97]. Bacterial transcriptome analysis found a decrease in transcript levels of a subset of chromosomal genes in a naturally occurring plasmid-free strain of* Ct*, demonstrating that the plasmid is a transcriptional regulator of virulence-associated genes [125]. Maintenance of the plasmid at the relatively low copy number of 5-6 plasmids per genome [143] carries inherent risk of plasmid-loss during cell partition [144], but naturally occurring plasmid-free strains are rare [145–147]. This suggests that 5-6 plasmid copies may maximize infectivity or intracellular survival whilst provoking minimal host immune response. Recent host transcriptome studies suggest that the plasmid may stimulate host cell expression of PD-L1, which induces programmed cell death in immune effector cells [48, 148]. The plasmid is clearly strongly selected for during successful infection or transmission [142] and recent studies have shown that a plasmid-cured* Ct* strain is less virulent in ocular infection of macaques and can function as a live attenuated vaccine [49].

The ability to obtain WGS data directly from clinical samples enables a genome-wide approach to investigate associations with disease phenotype and chlamydial load in naturally occurring* Ct* infections. Pathogen GWAS studies are a powerful tool to assess the association between a SNP or locus and defined phenotypes. It has also been shown to be a robust approach for discovering novel loci of interest in other bacterial pathogens [149, 150]. A small number of bacterial GWAS have revealed pathogen genetic associations with host adaptation in* Campylobacter jejuni* and* Escherichia coli* [151], drug resistance in* Mycobacterium tuberculosis* [152], methicillin-resistant* Staphylococcus aureus* (MRSA) [153], and* Streptococcus pneumoniae* [154] and resulted in the identification of virulence loci in methicillin-resistant* Staphylococcus aureus* [149]. Rapid advances in WGS techniques will enhance our understanding of chlamydial biology and diseases pathogenesis.

## 5. Bacterial Ecology of the Conjunctiva and Trachoma

Persistent, severe inflammation is associated with conjunctival scarring yet* C. trachomatis* is rarely detected in the eyes of adults with disease signs [7]. This has led to the suggestion that the inflammation seen in trachomatous disease is driven, at least in part, by nonchlamydial pathogens and there is now a growing evidence to support this hypothesis [155].

Studies carried out in The Gambia in the early 2000s found that whilst viable bacteria, including* Staphylococcus aureus* and* Streptococcus* species, could be cultured from the eyes of healthy controls, bacterial infection was more frequently identified in the eyes of cases with trachomatous trichiasis [156];* Streptococcus pneumonia* was the dominant pathogen isolated. In trachoma endemic communities in Tanzania, individuals with TS have been shown to be more frequently infected with* Streptococcus pneumoniae*,* Haemophilus influenzae*, coagulase-negative* Staphylococcus*, and* Corynebacterium* species than individuals with normal conjunctivae [155]. Similar findings have been reported in Ethiopians where comparison of the bacterial flora of conjunctivae with trachomatous trichiasis versus trachomatous scarring revealed increased frequency of isolation of* Streptococcus pneumoniae*,* Haemophilus influenzae*, coagulase-negative* Staphylococcus*,* Corynebacterium*, and viridans streptococci species [157].

More recent studies have examined the presence of viable bacteria in the eyes of children in regions of Tanzania and The Gambia where there is a low prevalence of clinical signs of active trachoma with correspondingly low levels of ocular* C. trachomatis* infection. In both studies, children with TF were more likely to have nonchlamydial bacteria in their eyes, namely,* Streptococcus pneumoniae* or* Haemophilus influenzae*, than children who had no clinical signs of disease [158, 159]. In the Tanzanian studies, culture of these nonchlamydial bacterial infections was associated with increased host conjunctival expression of* IL17A*,* CXCL5*,* CCL18*, and* KLRD1* [37]. Of these,* IL17A*,* CXCL5*, and* CCL18* were also associated with follicular and papillary inflammation.

The suggestion that nonchlamydial bacterial infection of the eye contributes to the maintenance of an inflammatory state that drives the scarring process is further supported by immunological studies. In Tanzania, a study of individuals with TS in the absence of TT found that nonchlamydial bacterial infection was associated with increased expression of* INDO*,* S100A7*,* DEFB4A*, and* MMP12* and decreased expression of* MMP10*,* SPARCL1*, and* CFH* [38]. Clinical inflammation, which is consistently identified as a major risk factor for progressive scarring, was associated with increased* INDO*,* IL1B*,* DEFB4A*,* CXCL5*,* MMP7*,* MMP9*,* MMP12*, and* CD83* and decreased* MMP10*,* SPARCL1*, and* CFH*. Data from longitudinal studies on recurrent trichiasis in The Gambia have indicated that bacterial infection, particularly with* Streptococcus pneumonia*, and clinical inflammation are associated with recurrence following surgery [160]. Clinical inflammation and nonchlamydial bacteria were also associated with increased expression of* IL1B* and* MMP9* in this cohort [161].

The isolation, often at high loads, of bacteria such as* Streptococcus pneumoniae* from the eyes of individuals with trachoma may suggest that colonization exacerbates clinical signs of the disease or, alternatively, that those with conjunctival inflammation are more susceptible to colonization and conjunctival scarring. Findings indicating increased numbers of what are largely regarded as commensal bacteria (such as coagulase-negative staphylococci and* Corynebacterium* species) in trachomatous eyes are also of particular interest, as alterations or dysbiosis of the microbiota could be a contributing factor to disease.

The study of the “normal” or indigenous microbiota that colonizes the human body has seen huge advances in the past decade with the application of NGS techniques that enable the identification of viral, fungal, and bacterial species. These methods have been particularly useful in the identification of noncultivable bacteria directly from clinical samples. These techniques have been perhaps best utilized to describe the microbiota of the human gastrointestinal tract and, in particular, changes in the microbiota associated with inflammatory disease including inflammatory bowel syndrome and Crohn's disease. Biopsied intestinal tissue that is inflamed tends to have reduced bacterial species diversity and altered species composition as compared to tissue taken from healthy controls [162–164]. The species composition of the microbiota has also been found to play an important role in the health of the epithelial barrier via induction and maintenance of IL-17, IL-22, and regulatory T cells [165–167].

In the female genital tract, dysbiosis of the microbiota (characterized by decreased numbers of lactobacilli and polymicrobial overgrowth of anaerobic bacteria) is associated with bacterial vaginosis. This and other alterations in the vaginal microbiota are thought to be risk factors for reduced fertility [168]. While the mechanism behind this is not yet clear, bacterial vaginosis has been associated with elevated cervical levels of IL-1*β* and IL-8, leading to the suggestion that the induction of proinflammatory cytokines by the altered vaginal microbiota is an unrecognized cause of infertility [169].

At present, the metagenome of the ocular surface has not been characterized and, to date, only a handful of studies have applied NGS techniques, namely, deep-sequencing of the 16S rRNA gene, to the characterization of the conjunctival microbiota. A study of four healthy American volunteers described a “core” conjunctival microbiota consisting of* Pseudomonas*,* Propionibacterium*,* Bradyrhizobium*,* Corynebacterium*,* Acinetobacter*,* Brevundimonas*,* Staphylococcus*,* Aquabacterium*,* Sphingomonas*, and* Streptococcus* species [170]. In contrast, the core members of the conjunctival microbiota of a West African population were identified as* Corynebacterium*,* Streptococcus*,* Propionibacterium*,* Bacillus*, and* Staphylococcus* species [171, 172]. Amongst ophthalmologists and microbiologists, these results have been questioned since the traditional view regards the ocular surface as a sterile site of low biomass. This view is not confined to those studying the ocular surface; there are many parallels with those investigating the lung microbiome where there was initial resistance to the concept that the lung was not a “sterile” environment but in fact that healthy lung harbors a complex community of bacteria [173].

Later studies on the conjunctival microbiota in Gambians with trachoma used a case-control study design with and without trachomatous disease (TF, TS, and TT) [172]. In common with other pathologies of the mucosal epithelia, this study found a disequilibrium of the normal flora, as defined by the decreased richness (number of genera) and diversity (number of genera and their abundance within the community) associated with clinical signs of TS and TT when compared with those with healthy eyes [172]. The decrease in bacterial diversity seen in individuals with TS was largely driven by an increase in* Corynebacterium* species in those with trachomatous disease, which is consistent with earlier studies characterizing the bacterial profile of the eye using culture techniques. The ocular microbiota of children was found to have greater richness and diversity in comparison to that of adults. While few significant differences were found in the microbiota of children with follicles versus normal controls, the microbiota of one child with clinical inflammation was dominated by* Haemophilus* species again pointing to the potential role of nonchlamydial bacteria in driving inflammation.

One drawback of the 16S rRNA gene sequencing approach, particularly in low biomass samples such as conjunctival swabs, is the potential for contamination from environmental sources and the inability to distinguish between viable and nonviable bacteria. Low biomass samples also currently preclude WGS approaches that would facilitate more a complete characterisation of the bacterial species and the full metagenome (archaea, fungi, and viruses). Using 16S-amplicon sequencing, it is difficult to distinguish those bacteria that actively colonize the conjunctival surface from those that are transiently introduced into this niche [172]. Primer-bias, which can lead to misrepresentation in the population [174], and bias introduced by sequencing depth, which limits the ability to characterise those bacteria that comprise the minority of the population [175], are also limitations of the 16S sequencing approach. As a result, there have been attempts to develop methods that might distinguish viable from nonviable bacteria before entry into the sequencing pipeline, such as the introduction of a DNase I predigestion [176], or the use of community transcriptomics to identify expressed bacterial components.

Culturomics is a relatively new field that couples high-throughput culture with matrix-assisted laser desorption ionization time-of-flight mass spectrometry (MALDI-TOF MS) identification and is a technique that may provide further means of addressing some of these issues. Studies of the gut microbiota have developed culturomics to identify bacterial species never before associated with the human gastrointestinal tract as well as novel species not previously described [177, 178]. As this technique targets viable cells, it is less prone to artefacts resulting from bacterial DNA contamination. The technique has also been used to establish cell-free* Chlamydia trachomatis* culture, using radiolabelled amino acids to demonstrate metabolic activity [99]. Culturomics therefore offers the potential to close the gap between 16S-amplicon sequencing and traditional microbiological culture [177, 179].

New applications of NGS continue to evolve in infectious disease immunology and the study of the role of the microbiota and specific microorganisms. One such technique is IgA-SEQ, a method that utilizes flow cytometry based sorting to separate bacteria coated with IgA from noncoated bacteria in a complex sample. The sorted fractions are then analysed by 16S-amplicon sequencing to identify the bacteria. In two recent studies, IgA-coated intestinal bacteria identified from human volunteers suffering inflammatory bowel disease and impaired gut-barrier function were cultured. These cultures were then able to induce intestinal inflammation in germ-free mice [180, 181]. These findings suggested that those bacteria that are able to penetrate the mucin barrier are more likely to stimulate an immune inflammatory response. The IgA-SEQ method thereby provides an elegant means of identifying and isolating those bacteria within the microbiota that drive disease.

The application of sophisticated techniques to the study of the conjunctival microbiota may offer better opportunities to distinguish bacterial colonizers from bystanders as well as identifying those bacteria that stimulate the immune responses that drive inflammation and the scarring process. The analysis of metagenomic data alongside host transcriptome arrays may also aid in this search; unraveling the interactions between the conjunctival microbiota and the gene expression networks and pathways of inflammation that lead to fibrosis is key to further understanding of microbiota-host interactions in scarring disease.

## 6. Epigenetics and Chlamydial Infection

One possible explanation for the chronic inflammation and continued presence of active disease signs in populations once ocular* Ct* infection has been controlled is epigenetic change of the host cell state. “Epigenetics” describes changes in gene expression that are heritable through cell division, without alteration to the DNA sequence itself. These changes can be applied directly to DNA (CpG methylation), to the proteins DNA is packaged around (chromatin modifications such as histone methylation or acetylation), and to the transcribed message (microRNA mediated silencing).

Bacteria and infection-induced inflammation are both known to induce epigenetic changes in host cells, particularly to genes in the MAPK, NFkB, and IFN*γ* signaling pathways, all of which are integral to the immune response (reviewed in [182]).* Helicobacter pylori* induces genome-wide aberrant hypermethylation of DNA in the gastric mucosa of pediatric and adult samples [183]. In particular, methylation of E-cadherin (and its downregulation, which is a characteristic of epithelial-mesenchymal transition (EMT), discussed further below) is induced by* H. pylori* infection via IL-1*β* stimulation of NFkB, resulting in nitric oxide production and activation of DNA methyltransferases [184]. In a similar manner, the Bacille Calmette-Guerin (BCG) vaccine induces long-lasting and nonspecific increases in cytokine expression in human monocytes via NOD2-dependent histone methylation of H2K4me3 [185].* Chlamydia* encode SET (suppressor of variegation-enhancer of zeste-trithorax) domain proteins, which function as methyltransferases [186].* Ct* encodes nuclear effector (NUE), a SET domain histone methyltransferase which is secreted through the T3SS and shows activity toward histones H2B, H3, and H4 in the host cell nucleus [187]. As of yet, the targets and function of NUE in the host remain to be identified. Humphrys and colleagues found that a number of host genes encoding histones were differentially expressed at 1 hour after infection, perhaps reflecting an early modulatory effect of* Ct* infection on the host epigenetic profile [188]. It is tempting to speculate that* Ct* stimulation of pathogen recognition receptors may induce CpG or histone methylation changes, or in fact that* Ct* may directly induce host histone changes via NUE, causing an abnormal state of chronic inflammation in the conjunctival epithelium.

## 7. The Role of Small RNAs in Chlamydial Disease

Only 20% of transcription across the human genome is associated with protein-coding genes [189] and it has become clear that many of the non-protein-coding RNA species that are transcribed have important regulatory roles. These noncoding RNA species include microRNA (miRNA), short interfering RNA (siRNA), piwi-interacting RNA (piRNA), small nucleolar RNAs (snoRNA), other small RNAs, and long noncoding RNA (lncRNA (>200 nucleotides)).

miRNA encoding sequences make up only around 2% of the human genome but are estimated to regulate >60% of protein-coding genes [190]. miRNA (miR) are short (18–22 nucleotides) single stranded sequences of RNA. miR regulate gene expression through binding to complementary messenger RNA (mRNA) sequences in the cytosol in association with Argonaute proteins, forming a RNA-induced silencing complex (RISC), leading to inhibition or degradation of the target mRNA [191]. The “seed sequence” of the miR, nucleotides 2–7 from the five prime ends, guides target selection [192]. Half of known miR are found in polycistronic units and are expressed in parallel, often sharing structure and function [193, 194]. Due to flexibility in binding complementarity, an individual miR can target hundreds of different genes and a gene might be targeted by many different miR [194], forming a complex network of regulation. Abnormal expression of miR may occur by the same factors regulating expression of any gene, including epigenetic control of pre-miR transcription or SNPs in the miR coding sequence. miR expression can also be regulated at the posttranscriptional level (as pre-miR or mature miR), by RNA-binding proteins or by circular RNAs that can act as “sponges” [195–198]. Due to the far-reaching and complex roles of miR, a small change in expression can have a profound effect on tissue homeostasis.

miR are an important part of the host response to bacteria, both pathogenic and commensal. Inflammation must be tightly regulated; excess can lead to organ damage whereas insufficient inflammation may facilitate the dissemination of infection. Several miR are well characterized as having important roles in the immune response against bacteria (reviewed in [199–201]). miR-146 and miR-155 are upregulated in immune cells following infection with a range of bacteria, including* Helicobacter pylori*,* Salmonella enterica*,* Listeria monocytogenes*,* Francisella tularensis*, and* Mycobacterium* species. Both miR-155 and miR-146 function in negative feedback loops to prevent excessive inflammation by silencing targets in the TLR4 signalling pathway. miR-155 also maintains TNF expression and is essential for an appropriate adaptive immune response. Distinct patterns of miR expression are elicited by two strains of* Cm* that vary in virulence in the murine genital tract [202]. miR-223-3p and miR-18a-5p were induced in mice by both strains at 24 hours after infection. Interestingly, miR-155 expression was increased in response to avirulent* Cm* but not in response to the virulent strain of* Cm*. The failure to upregulate miR-155 in the virulent infection could indicate an absence of the negative feedback loop that is required to prevent excessive inflammation, leading to the increased pathology that was observed. Gupta and colleagues investigated miR expression following* Cm* infection in the murine genital model at a later time point. At 6 days after bacterial challenge, miR-125b-5p, miR-135a, miR-16, miR-214, miR-30c, miR-30e, miR-182, miR-183, and miR-23b were downregulated in the lower genital tract and miR-146 and miR-451 were upregulated [203]. These changes were not maintained at 12 days after infection. Knockdown of miR-125b-5p, miR-30c, and miR-182 led to a failure to control* Cm* infection and, in CD4^−/−^ mice, levels of miR-125b-5p, miR-182, miR-183, and miR-135 were upregulated relative to wild-type infected mice. miR-125b maintains the naivety of T cells and is downregulated upon their differentiation and maturation [204]. miR-125b also targets TNF and is thought to maintain the inactivity of macrophages in the absence of bacterial TLR stimulation [199]. miR-125b downregulation may therefore be required for an appropriate immune response. Igietseme and colleagues have shown that, upon urogenital infection of mice with a virulent LGV* Ct* strain (L2), murine miR-21, miR-103, miR-107, let-7i, and miR-92b were downregulated in the oviducts, though the time point at which these changes in expression are observed is unclear [205].

Overexpression of miR-146a in psoriatic skin lesions has been linked to a SNP in the miR-146 gene in a large cohort of Chinese patients [206]. Wang and colleagues looked at the association of polymorphisms in miR-146a and the NRLP3 inflammasome in association with susceptibility and severity of urogenital* Ct* infection in two cohorts of Dutch and Finnish women [207]. A SNP in NLRP3 was found to associate with lower abdominal pain in* Ct* positive women; however, no link with miR-146a was found. Our previous work has identified that miR-147b is upregulated in scarring and inflammatory trachoma and miR-1285 is upregulated in scarring and inflammation when compared to scarring alone [208]. miR-147b acts in a similar manner to miR-146a; it is induced by TLR signaling and prevents excessive inflammation in murine macrophages [209] and is thought to have a homologous role in humans [210]. The upregulation of miR-147b in diseased individuals could represent repeated TLR stimulation, perhaps by an abnormal microbiome. miR-1285 has conflicting roles in regulation of the cell cycle [211, 212]. Abnormal regulation of inflammation and cell cycle could drive the chronic inflammation and fibrotic proliferation associated with trachomatous pathology.

Transcriptome arrays comparing gene expression at various stages of trachomatous disease have found that many thousands of genes are differentially regulated [34, 36]. In an attempt to reduce this complexity, we used a data mining approach (MSigDB) and found miR that were enriched for targets within lists of differentially regulated genes of four trachoma transcriptomes (Table 1). Of interest are the miR with known roles in the regulation of EMT, which we discuss further below. A number of miR that have been identified as differentially expressed in* Ct* and* Cm* infection are enriched for targets in these datasets, particularly in the comparison of active disease with* Ct* infection against controls, which is closest biologically to these murine models. Many of the miR were predicted to have roles regulating cell proliferation and apoptosis. This could be reflective of* Ct* preventing host cell apoptosis to maintain the intracellular niche or T cell and fibroblast proliferation contributing to inflammation and fibrosis; however, a drawback of using data mining techniques is the relative abundance of cancer-related miR associations and pathways that dominate the literature. Pathogens and commensals express their own regulatory RNAs and it is emerging that these can be translocated into host cells to subvert or manipulate the response. Several DNA viruses express miR that target viral and host mRNAs to enhance their own success and some express virulence factors that manipulate host miR levels (reviewed in [213, 214]).* Mycobacterium tuberculosis* secreted effector ESAT-6 downregulates let-7f in macrophages, releasing expression of A20, which is a negative feedback regulator of the NFkB pathway [215]. This results in a reduction of inflammatory cytokine production and increased* M. tuberculosis* survival. miR from the parasitic nematodes* Heligmosomoides polygyrus* and* Litomosoides sigmodontis* have recently been found in the serum of mice and in discrete parasite-secreted exosomes, resulting in systemic immune regulation and suppression following uptake by host cells [216]. Little is known about bacterial miR and their ability to modulate host responses.* Mycobacterium marinum* expresses a candidate miR (23 nt long) that can be bound by host RISC and silence an mRNA target, though a functional role has not yet been identified [217].* Ct* does not appear to express any authentic miR species in human cells, as defined by association with RISC proteins and a length of 22 ± 2 nt [217].* Ct* does, however, encode a number of noncoding RNAs with distinct expression patterns throughout the developmental cycle, one of which regulates the transcription of a* Ct* gene [218, 219]. Some lncRNA encoding sequences are located on the* Ct* plasmid, a known virulence factor [220]. No effect on chlamydial gene transcription was identified by “knockout” of one of these plasmid-encoded miR, but an effect on host transcription was not examined [97].

## 8. Epithelial-Mesenchymal Transition and Chlamydial Disease

EMT is a reversible process by which epithelial cells differentiate into mesenchymal cells. Currently 3 types of EMT are recognised. In type-I EMT, epithelial cells differentiate into mesenchymal cells to migrate around the growing embryo during fetal development. These mesenchymal cells can then transform back into epithelia through the reverse process of mesenchymal-epithelial transition (MET) to establish new distant sites of epithelial tissue. Type-II EMT is associated with inflammation-induced tissue fibrosis and type-III EMT occurs in cancer when epithelial tumors metastasize. It is thought that the 3 types of EMT have similar stimuli, signaling cascades, and regulation [221, 222]. Endothelial cells also differentiate into mesenchymal cells when subjected to the same stimuli (EndMT) and can contribute to cardiac fibrosis [223].

Type-II EMT (EMT-2) occurs in response to inflammation and can lead to loss of organ function through pathological fibrosis. Inflammatory cytokines, growth factors, and MMPs activate a chain of transcription factors that cause downregulation of epithelial characteristics in cells and gain of mesenchymal properties. The basement membrane is degraded by MMPs, allowing differentiating cells to migrate into the interstitial tissue [224]. New mesenchymal cells can then acquire a myofibroblast phenotype [225], secreting collagen 1 and expressing *α*-smooth muscle actin and therefore contributing to extracellular matrix deposition and “wound” contraction. EMT-2 is known to contribute to the pathology of many fibrotic diseases, including kidney fibrosis [225], idiopathic pulmonary fibrosis [226], and cardiac and liver fibrosis [223, 227]. In a murine model of liver fibrosis, up to 45% of fibroblasts were found to have originated from hepatocytes [227]. Evidence of EMT has been shown both in acute wound healing and in chronic fibrotic (hypertrophic) scars where there is also evidence of unresolved inflammation [228]. EMT-2 ceases when inflammatory stimuli stop; therefore it appears that EMT-2 only become pathological in an environment of chronic inflammation. Having said that, EMT cells can be resistant to apoptosis (reviewed in [229]), possibly arising from the need for resilient cells to seed new sites of epithelial tissue in embryonic development. In addition to resisting apoptosis, EMT cells can acquire stem-cell-like properties allowing self-renewal [230–232], a property which is known to play a role in various cancers and which could contribute to the chronic fibrosis that is observed in trachoma. EMT-activating transcription factor ZEB1 represses expression of the stemness-inhibiting miR-200 family, therefore releasing expression of stem-cell factors and leading to EMT and self-renewal. From the evidence of our* in silico* analysis (Table 1), we suggest that the miR-200 family may be associated with trachoma.

Epigenetic changes can fix the expression of genes that induce EMT, contributing to chronic fibrosis and cancer. TGF*β*-induced EMT is associated with global loss of heterochromatin mark H3K9Me2 and gain of euchromatin marks H3K4Me3 and H3K36Me3 [233]. In cancer-related EMT, hypermethylation of E-cadherin is found [234, 235]. SNAIL, a transcription factor associated with EMT, is thought to contribute to this effect by recruiting DNA methyltransferase-1 to the E-cadherin promoter [236] and has been shown to modify the chromatin structure at the site through recruitment of the Sin3A/histone deacetylase 1 (HDAC1)/HDAC2 complex [237].

Primary hepatocytes infected with hepatitis C virus* in vitro* have been shown to upregulate biomarkers of EMT [238]. LPS-treated intrahepatic biliary epithelial cells upregulate TGF*β*1* in vitro* and stimulate EMT through the TGF*β*1/SMAD2/3 pathway [239]. EMT-2 has also been observed during herpes virus infection* in vitro* and* in vivo* and following* E. coli* infection* in vitro* [240, 241].* Helicobacter pylori* virulence factors VacA and CagA disrupt epithelial cell tight junctions and polarity [242–244] and upregulation of EMT transcription factors and biomarkers in gastric cell lines requires both the* cag* pathogenicity island and NFkB activation [245, 246]. EMT can thus be stimulated indirectly through inflammatory signals or directly by pathogens.* Ct* infected epithelial cells in* ex vivo* fallopian tube tissue show phenotypic changes characteristic of EMT, such as decreased polarity and cell adhesion [247]. The WNT pathway was upregulated and *β*-catenin was recruited to the chlamydial inclusion. Loss of epithelial cell-cell adhesion, N-cadherin/*β*-catenin complex formation, and recruitment of *β*-catenin to the chlamydial inclusion have also been observed* in vitro* [248]. Nectin-1, an integral molecule of epithelial cell tight junctions and adherens junctions, was downregulated at the posttranscriptional level by 85% in* Ct* infected HeLa cells, which was dependent on live infection [249]. Disruptions to key components of the WNT and Notch signaling pathways, intercellular junctions and adhesion, and cytoskeletal remodeling have been identified by RNA-sequencing as early as one hour after infection of HeLa cells with serovar E [188]. Transcriptomic profiling of TT and subsequent gene-set enrichment analysis has also shown significant enrichment in members of the WNT pathway [36, 250]. These data therefore support a role for WNT signaling in both active infection and later stages of chlamydial disease. Given that WNT signaling stimulates EMT* in vitro* [251] and the Akt/*β*-catenin pathway mediates EMT activation following HCV and* H. pylori* infection [238, 244], it is therefore tempting to speculate that* Ct* induces EMT-2 in the conjunctival epithelium, either directly or via* Ct*-induced inflammation.

## 9. Conclusions

The development of scarring trachoma represents a complex multigenic system where each host factor contributes a small protective or deleterious risk. Interaction of each of these factors adds a further layer of complexity to understanding of the disease process. Outside of host genetic factors, balance or dysbiosis of the ocular surface microbiome coupled to* Ct* genetic variation and environmental risk factors each contributes to a multilayered disease network that determines protection or pathology. Excessive or uncontrolled prolonged inflammation is the main risk factor for pathology. Genes and pathways that are dysregulated in inflammation, infection, and scarring have been identified, but clear causative links between inflammation and scarring remain elusive. To address this, we need* in vitro* studies and longitudinal clinical studies of infection, inflammation, and progressive scarring in childhood when scarring evolves. Unlike human sexually transmitted chlamydial infections, studies such as these are still possible in trachoma endemic populations. The current trend [252, 253] and emerging toolkit [254–263] for using a systems-wide or systems biology analysis of data promise to look beyond single markers towards entire molecular and cellular pathways across multiple layers of disease. The ultimate goal of the systems approaches should be to define a model that integrates data from these multiple layers including the host genetic background, composition of the microbiome, host response (transcriptome, proteome, and epigenome), and pathogen variation [188, 264] in order to explain the route to scarring or protection.

## Figures and Tables

**Table 1:** miR predicted to regulate differentially expressed transcripts from four array datasets.

miR functional categories	MsigDb predicted miR based on differentially regulated mRNA transcripts			
	(FC > 1.5 Adj. *P* < 0.01) from 4 trachoma transcriptomes			
	Active disease with *Ct* infection (GSE20436)	Active disease (GSE20430)	Trachomatous scarring disease with inflammation (GSE24383)	Trachomatous trichiasis with inflammation (GSE23705)
Inflammation/infection	**let-7 family **[205]		miR-511	**let-7 family** [205]
	**miR-125a/b** [203]			miR-19a/b
	miR-19a/b			miR-224
				miR-29a/b/c

EMT/fibrosis	miR-200b/c, miR-429			miR-200b/c, miR-429
	miR-506			miR-29a/b/c
				miR-506
				miR-520d

Cell cycle/cancer	**let-7 family** [205]	miR-518a-2	miR-128a/b	**let-7 family** [205]
	miR-124a	miR-186	**miR-21** [202, 205]	miR-19a/b
	**miR-125a/b **[203]****	miR-130a/b, miR-301	miR-26a/b	miR-22
	miR-15 family		miR-519a/b/c	miR-224
	miR-17-5p, miR-20a/b, miR-106a/b, and miR-519d			miR-25, miR-32, **miR-92**, miR-363, and miR-367 [205]
	miR-19a/b			miR-516-3p
	miR-218			miR-519a/b/c
	**miR-23a/b **[203]****			miR-520d
	**miR-30a/b/c/d/e-5p **[203]****			
	miR-519a/b/c			
	miR-524			
	miR-9			

Other	miR-130a/b, miR-301		miR-153	miR-27a/b
				miR-516-5p

Phenotype comparisons are as follows: normal healthy (N) children aged 1–9 versus those with active trachoma (TF) and *Ct* infection (GSE20436); children aged 1–9 with TF (with or without *Ct* infection) versus N (GSE20430); N adults versus adults with trachomatous scarring and clinical inflammation (TSI) (GSE24383); and N adults versus adults with trichiasis and clinical inflammation (TTI) (GSE23705). Enriched miR are grouped into functional categories based on well-characterized roles in the literature. References show published studies that identify miR in bold as differentially expressed in chlamydial infection.
